# Antigen-specific T cells conditioned with MDSC display a surprising increased anti-tumor activity after adoptive T cell-based immunotherapy

**DOI:** 10.1186/2051-1426-3-S2-P413

**Published:** 2015-11-04

**Authors:** Patrick Raber, Paul Thevenot, Rosa Sierra, Paulo C Rodriguez

**Affiliations:** 1 LSU-HSC, New Orleans, LA, USA

## 

The success of adoptive T cell-based immunotherapy (ACT) in cancer is limited in part by the accumulation of myeloid-derived suppressor cells (MDSC), which block several T cell functions, including proliferation and expression of effector mediators. Paradoxically, inhibition of CD8^+^ T cell differentiation during the pre-ACT phase also showed to increase anti-tumor efficacy. Thus, we aimed to determine the effect of MDSC on T cell differentiation and on ACT efficacy after *in vitro* conditioning of CD8^+^ T cells. Results indicate that MDSC block the differentiation of CD8^+^ T cells into effector cells, without altering their activation status, production of IL-2, or signaling through the T cell receptor. Moreover, culturing of CD8^+^ T cells in the presence of MDSC resulted in an increased ACT anti-tumor activity and elevated frequency and IFNγ production after transfer onto tumor-bearing mice. Additional findings confirmed that undifferentiated CD62L^+^ T cells mediated the enhanced anti-tumor activity triggered by MDSC-exposed T cells. Mechanistic studies showed that MDSC restricted *de novo* protein synthesis and activity of mechanistic target of rapamycin (mTOR) in T cells. Silencing of the negative mTOR regulator tuberous sclerosis complex 2 restored mTOR activity in T cells co-cultured with MDSC, but resulted in T cell apoptosis. Thus, our results indicate that culturing of CD8^+^ T cells in the presence of MDSC improves their anti-tumor efficacy, frequency, and function after ACT possibly through inhibition of mTOR signaling. Continuation of this research will enable the development of novel strategies to enhance the efficacy of ACT in cancer.

